# Structure of the SLy1 SAM homodimer reveals a new interface for SAM domain self-association

**DOI:** 10.1038/s41598-018-37185-3

**Published:** 2019-01-10

**Authors:** Laura Kukuk, Andrew J. Dingley, Joachim Granzin, Luitgard Nagel-Steger, Pallavi Thiagarajan-Rosenkranz, Daniel Ciupka, Karen Hänel, Renu Batra-Safferling, Victor Pacheco, Matthias Stoldt, Klaus Pfeffer, Sandra Beer-Hammer, Dieter Willbold, Bernd W. Koenig

**Affiliations:** 1 Institute of Complex Systems, Strukturbiochemie (ICS-6), Forschungszentrum Jülich, 52425 Jülich, Germany; 2 Institut für Physikalische Biologie, Heinrich-Heine-Universität Düsseldorf, Universitätsstraße 1, 40225 Düsseldorf, Germany; 3 Institut für Makromolekulare Chemie, Albert-Ludwigs-Universität Freiburg, Stefan-Meier-Straße 31, 79104 Freiburg, Germany; 4 Institut für Medizinische Mikrobiologie und Krankenhaushygiene, Heinrich-Heine-Universität Düsseldorf, Universitätsstraße 1, 40225 Düsseldorf, Germany; 5 Institut für Experimentelle und Klinische Pharmakologie und Toxikologie, und Interfakultäres Zentrum für Pharmakogenomik und Arzneimittelforschung (ICePhA), Eberhard-Karls-Universität Tübingen, Wilhelmstraße 56, 72074 Tübingen, Germany

## Abstract

Sterile alpha motif (SAM) domains are protein interaction modules that are involved in a diverse range of biological functions such as transcriptional and translational regulation, cellular signalling, and regulation of developmental processes. SH3 domain-containing protein expressed in lymphocytes 1 (SLy1) is involved in immune regulation and contains a SAM domain of unknown function. In this report, the structure of the SLy1 SAM domain was solved and revealed that this SAM domain forms a symmetric homodimer through a novel interface. The interface consists primarily of the two long C-terminal helices, α5 and α5′, of the domains packing against each other. The dimerization is characterized by a dissociation constant in the lower micromolar range. A SLy1 SAM domain construct with an extended N-terminus containing five additional amino acids of the SLy1 sequence further increases the stability of the homodimer, making the SLy1 SAM dimer two orders of magnitude more stable than previously studied SAM homodimers, suggesting that the SLy1 SAM dimerization is of functional significance. The SLy1 SAM homodimer contains an exposed mid-loop surface on each monomer, which may provide a scaffold for mediating interactions with other SAM domain-containing proteins via a typical mid-loop–end-helix interface.

## Introduction

The multifunctional adaptor protein SLy1 (SH3 domain-containing protein expressed in lymphocytes 1), also known as SASH3 (SAM and SH3 domain containing protein 3), is exclusively expressed in lymphocytes and plays different roles in various lymphocyte subsets^1,2^. SLy1 is important for T and B cell development and proliferation, and for complete activation of adaptive immunity^3–5^. In natural killer (NK) cells, SLy1 acts as a ribosomal support protein facilitating ribosomal stability and in turn viability and activation of mature, peripheral NK cells^6^. Lack of SLy1 compromises NK-mediated immunosurveillance, reduces cytotoxicity toward malignant and non-malignant NK cell targets, and increases cancer susceptibility^6^. In T and B cells SLy1 is located in the cytoplasm and nucleus^1,3^.

SLy1 is 380 amino acids in length and contains a bipartite nuclear localization signal (NLS), a Src homology 3 (SH3) and a sterile alpha motif (SAM) domain (Supplementary Fig. S1), which makes SLy1 a prototypical adaptor protein. Murine and human SLy1 show 94% sequence identity, and the amino acid sequence of the SAM domain is identical in both proteins^1^. Both SH3^7^ and SAM^8,9^ domains have been shown to mediate protein-protein interactions in other proteins^9–11^. However, the specific functional roles of the SLy1 SAM and SH3 domains remain unresolved. SAM domains contain ~70 amino acids and share a common structural motif of five helices^8,9^. They are found in a multitude of proteins in organisms ranging from yeast to mammals and are involved in diverse biological functions including transcriptional and translational regulation, cellular signalling, and regulation of developmental processes^9^. SAM domains connect proteins to other proteins via SAM domains or non-SAM interaction motifs (e.g., PDZ and SH2 domains). A subclass of SAM domains specifically binds RNA^12^ and function as an RNA recognition element^13^. In addition, particular SAM domains bind to lipid membranes^14,15^.

Many SAM domains self-associate to form homodimers^16–18^, closed oligomeric structures^19^ or extended polymers^20,21^. Published equilibrium dissociation constants for SAM homodimers range between 0.5 and 5 mM for Ste11 SAM^18^ and EphA2 SAM^16^, indicating weak affinity. Homopolymeric SAM structures with demonstrated biological relevance form left-handed helices with six SAM monomers per turn^21^. Neighbouring monomers join in a head-to-tail fashion utilizing two opposite interfaces on the compact monomer structure referred to as end-helix (EH) and mid-loop (ML) surfaces^20^. The stability of the fibrils depends on the affinity of the interacting EH and ML surfaces. Experimentally determined equilibrium dissociation constants for this interaction range from 1.7 nM for TEL-SAM fibrils^20^ to 11 µM for Yan SAM fibrils^22^. SAM domains can also specifically bind to other SAM domains to form heterodimers^22,23^ or heterooligomers^24^. Examples with dissociation constants in the low micromolar range have been reported for the heterodimers of Odin SAM1/Arap3 SAM^25^, Odin SAM1/EphA2 SAM^26^ and Ship2 SAM/EphA2 SAM^27^. Other pairs like Mae SAM/Yan SAM^22^ or CNK SAM/Hyp SAM^23^ show nanomolar affinity.

Self-association also plays a role in cellular signal transduction. The function of a number of adapter proteins involved in signalling like Grb2 (growth factor receptor binder 2) and 14-3-3 proteins relies on dimerization^28,29^. Moreover, immune adapter SLP-76 (Src homology 2 domain-containing leukocyte protein of 76 kDa) self-association in response to T-cell receptor ligation is mediated by its N-terminal SAM domain^30^. We hypothesize that dimerization of SLy1 is required for its biological function and that the SAM domain plays a role in this process.

In the current report, we show that the SAM domain of SLy1 self-associates to form a distinct dimer with an affinity much stronger than reported previously for homodimerization of other SAM domains. Structure determination reveals that this SAM domain forms a novel type of dimer interface that is incompatible with homooligomerization. The stability of the homodimer is sensitive to the presence of amino acids flanking the SAM domain. Our findings on SLy1 SAM self-association suggest that SLy1 functions as a dimer.

## Results

Three SLy1 SAM domain constructs were produced successfully in *E.coli* as glutathione S-transferase (GST) fusion proteins, released by PreScission cleavage, and purified to homogeneity. The first variant is referred to as SAM_wt_ and contains the SAM domain (P254–Y316), the five residues D317-E321 succeeding it in SLy1, and an N-terminal glycine from the tag (Supplementary Fig. S2). SAM_C_ differs from SAM_wt_ by an S320C amino acid exchange, which is located outside the predicted SAM domain. The slightly longer SAM_lg_ comprises residues G249-E321 of SLy1 preceded by a glycine-proline tag. Typical yields of the purified SAM variants were 5–8 mg per litre of culture medium.

### The SLy1 SAM domain exists in a monomer-dimer equilibrium

**Figure 1:**
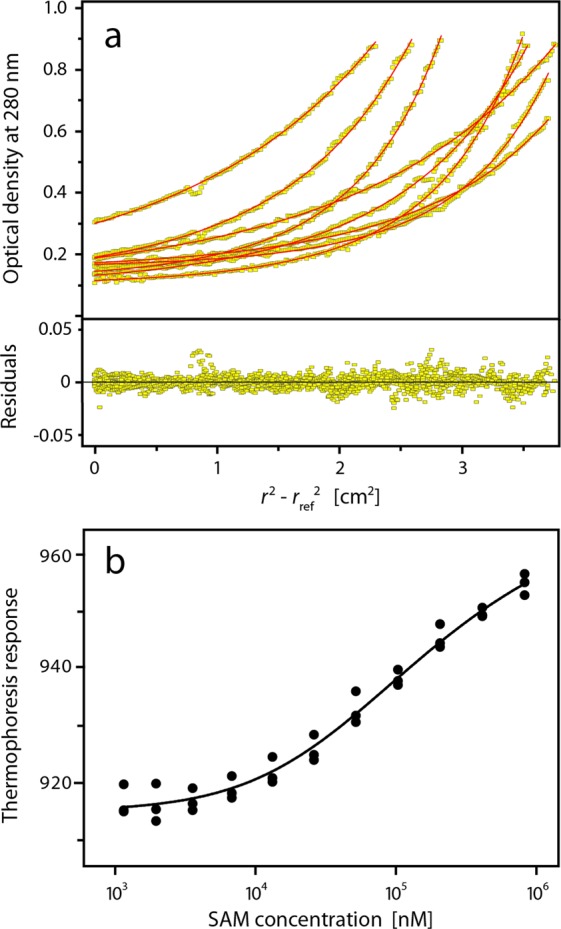
Analysis of the SLy1 SAM_wt_ monomer-dimer equilibrium. (**a**) Sedimentation equilibrium experiments were performed using samples with different SAM_wt_ concentrations (60, 120, and 300 µM) at different speeds. Data were fit globally with a monomer-dimer model. The upper panel shows concentration profiles recorded after establishment of equilibrium between sedimentation and back diffusion and the calculated concentration distributions (red lines) based on a monomer-dimer equilibrium model. The global fit gives a *K*_d_ = 117 (33, 423) μM. The lower panel shows the residuals of the fit. (**b**) Binding isotherm from microscale thermophoresis data. The thermophoresis response of fluorescent labelled SAM_wt_ is dependent on the total concentration of SAM_wt_. The experimental data were fit to a monomer-dimer equilibrium model (solid line) with a *K*_*d*_ of (153 ± 25) μM.

The calculated molecular mass of SAM_wt_ is 7,929 Da. Size exclusion chromatography used in the purification of SAM_wt_ indicated that this protein oligomerizes, which was also supported by blue native PAGE experiments showing that SAM_wt_ is monomeric at two digit micromolar concentrations but monomer and a minor dimer fraction is observed at a gel loading concentration of 210 µM (see Supplementary Fig. S3). Thus, sedimentation equilibrium experiments were performed to characterize the SAM domain oligomerization in detail. An acceptable global fit of the data was achieved only with the monomer-dimer equilibrium model with low residuals throughout the radial concentration profile (Fig. 1a). The global fit of the sedimentation profiles resulted in a *K*_a_ = 8.5 (2.4, 30) × 10^3^ M^−1^, i.e., *K*_d_ = 117 (33, 423) µM, and a molecular mass of (8,040 ± 532) Da for the SAM_wt_ monomer. The numbers in parenthesis specify the 95% confidence interval. Both the one-component and the two-component models provided inferior fits with significantly larger residuals than the monomer-dimer equilibrium model. Microscale thermophoresis (MST) was used as an orthogonal method to measure the binding affinity of the SAM domain self-association (Fig. 1b). The experimental thermophoresis response curves could be fitted with a monomer-dimer equilibrium model, providing a dimer dissociation constant of *K*_d_ = (153 ± 25) μM.

### Cross-linking the SAM_C_ monomers quenches chemical exchange

Variation in the intensity and line-widths of resonances in the 2D ^1^H-^15^N HSQC spectrum of SAM_wt_ indicated the presence of chemical exchange, presumably because of the monomer-dimer equilibrium. 2D ^1^H-^15^N HSQC spectra were acquired at different SAM_wt_ concentrations. Overlay of the spectra showed that the chemical shift of resonances changed and the line widths of particular resonances showed strong line-broadening because of intermediate-to-fast exchange on the μs-ms timescale (Supplementary Fig. S4). Initial attempts to determine the three-dimensional (3D) structure of SAM_wt_ by NMR spectroscopy were unsuccessful because the chemical exchange prohibited the collection of reliable interproton distance restraints. In particular, the heteronuclear filtered NOESY data, which provide intermolecular distance restraints, suffered from a very low signal-to-noise ratio and showed only a limited number of cross correlations (Supplementary Fig. S5). The introduction of an intermonomer disulphide bond stabilized the native dimer state and eliminated the chemical exchange process, thereby facilitating structure determination by NMR spectroscopy. This was achieved by exchanging S320, which is located C-terminal to the predicted SAM domain, with cysteine, to give SAM_C_. 2D ^1^H-^15^N HSQC spectra of SAM_C_ in the reduced and oxidized state were similar, indicating that disulphide bond formation did not perturb the overall fold of the SAM domain (Supplementary Fig. S6). Weighted chemical shift mapping between SAM_wt_ and reduced-state SAM_C_ showed that the S320C exchange caused negligible chemical shift changes with only pronounced changes observed around the mutation site (Supplementary Fig. S7). In addition, the 2D ^1^H-^15^N HSQC spectrum of the disulphide-bonded SAM_C_ was also similar to the SAM_wt_ spectrum (Supplementary Fig. S7) with chemical shift differences for resonances of residues around the mutation site and a few larger than average chemical shift changes for resonances corresponding to residues further away from the S320C exchange, which are due to reduced mobility of the C-terminus and the difference in dimer population.

### NMR structure of the SAM_C_ homodimer reveals a new interface

Two- and three-dimensional NMR spectra of the cross-linked SAM_C_ dimer are well-resolved and show a single set of resonances corresponding to the amino acid sequence of the monomer, thus indicating a symmetric homodimer^31–33^. Near complete (97%) assignment of SAM_c_ resonances was achieved (Supplementary Table S8). A total of 5,026 partially assigned NOESY cross peaks were obtained from the five 3D NOESY spectra recorded. This number includes 286 cross peaks from the two double isotope-filtered NOESY spectra that provide intermonomer cross correlations exclusively. Fifty-eight backbone ϕ/ψ torsion angle pairs were derived from the chemical shift data using TALOS+^34^. In addition, 16 experimentally determined backbone H-bonds were used as restraints. Sidechain χ_1_ torsion angles of 24 residues were restrained to one of the staggered conformations (60°, 180°, −60°) ± 30° as determined by combined ^3^*J*_HαHβ_ and ^3^*J*_NHβ_ couplings analysis. Iterative NOESY data analysis and structure calculation by ARIA^35,36^ extracted 2,466 unambiguously assigned and 1,593 ambiguous distance restraints from the NOESY data (Supplementary Table S8). Among the uniquely defined restraints are 236 intermonomer and 406 long-range intramonomer distances.

**Figure 2:**
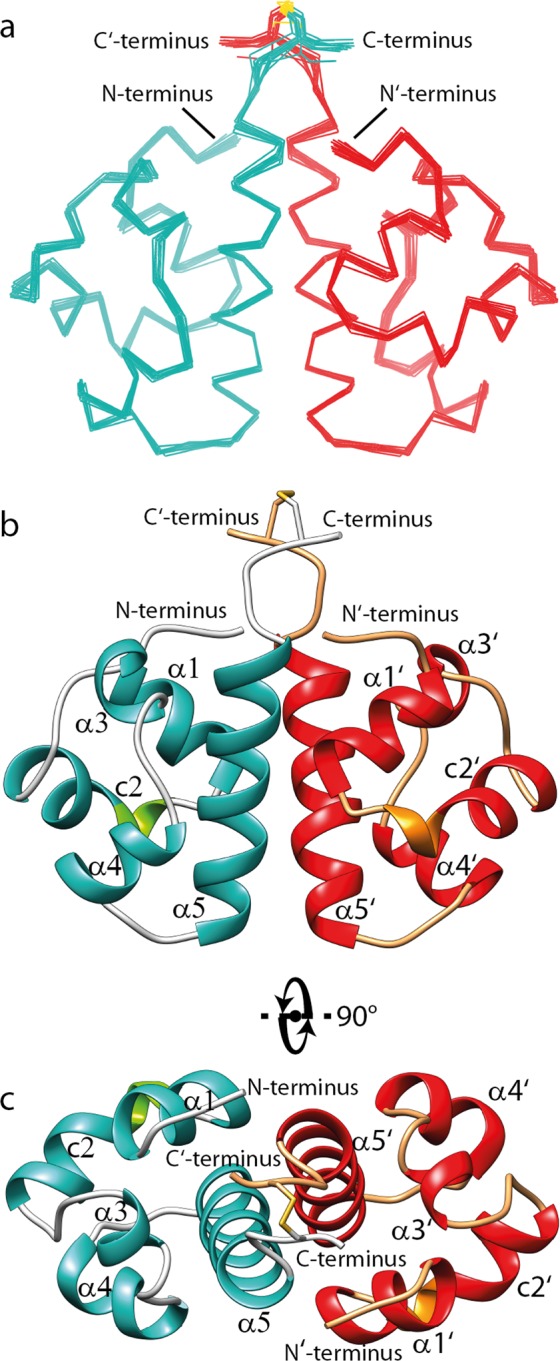
NMR solution structure of SLy1 SAM_C_ homodimer. (**a**) Superposition of the 15 lowest energy structures of the disulphide bond-stabilized homodimer of SAM_C_. The backbones of the monomers are coloured teal and red. (**b**,**c**) Ribbon representation of the structure closest to the average backbone structure (r.m.s.d. = 0.19 Å) of the ensemble. α-helices are shown in teal and red in each monomer, whereas the 3_10_-helix part in the composite helix c2 is shown in green and orange. Helices α5 and α5′ are in tight contact to form the major part of the interface. They run in a parallel fashion with an angle of ~−50° between their long axes. Helix α1 and the N-terminus of one monomer are in close proximity to the C-terminal region of helix α5′ of the opposing monomer, and also form part of the dimer interface.

Superposition of the final 15 lowest energy models of the SAM_C_ homodimer refined in an explicit water shell is shown in Fig. 2a. The coordinate root-mean-square deviation (r.m.s.d.) is 0.31 Å for the backbone heavy atoms and 0.57 Å for all heavy atoms. Structural statistics are presented in Supplementary Table S8. RPF analysis indicates excellent agreement between the experimental NOESY data and the calculated NMR structure of the SAM_C_ homodimer, and the high DP-score shows that the amount of data defines the structure accurately^37^. Superposition of the two monomers of any of the 15 SAM_c_ homodimer models yields a pairwise C_α_ r.m.s.d. of no more than 0.01 Å in line with a symmetric homodimer.

Each monomer in the NMR structure shows the typical five helix bundle of the canonical SAM domain fold (Fig. 2b,c)^38^. DSSP^39,40^ analysis of the SAM_C_ atomic coordinates suggested an H-bond pattern in agreement with four α-helices: α1 (L257–I264), α3 (L281–F284), α4 (E289–L295) and α5 (P300–D315). In addition, a kinked composite helix c2 (E267-L275) was identified consisting of a 3_10_-helix (E267–H269) and an α-helical segment (T270–L275).

**Figure 3:**
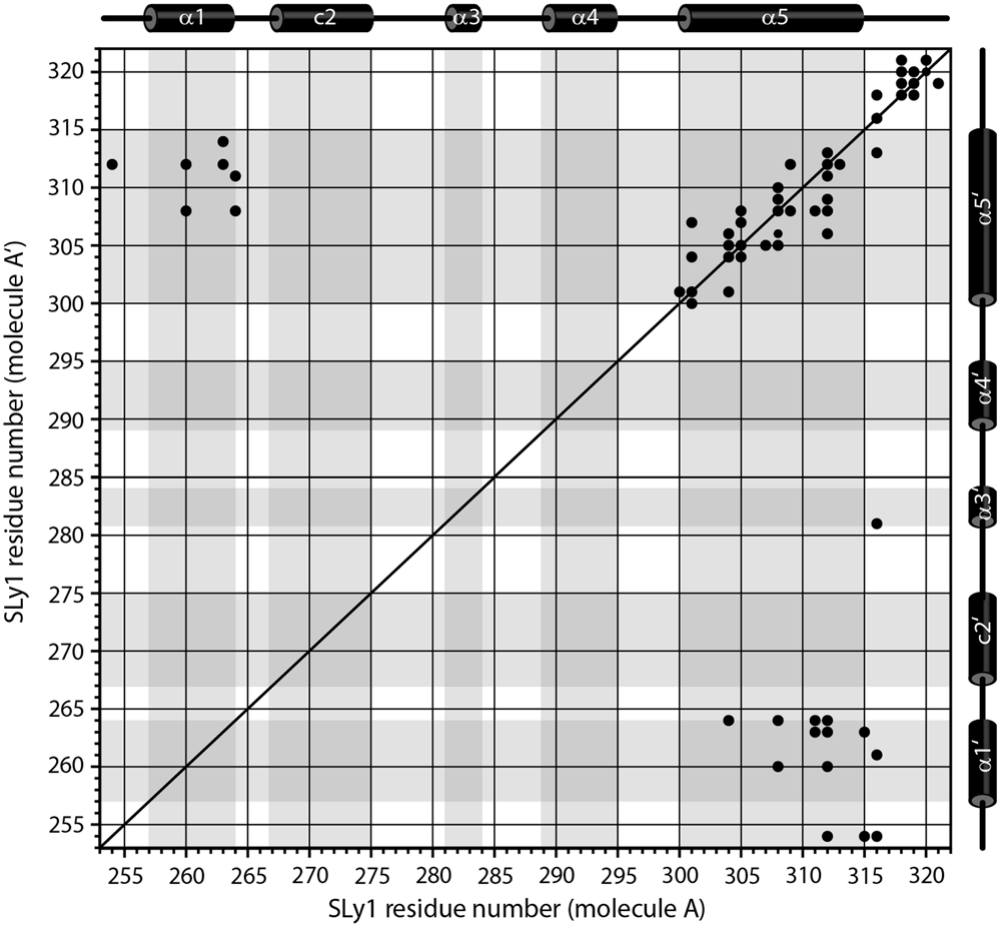
Intermonomer contact pattern in the SLy1 SAM_C_ homodimer. Intermonomer NOE cross correlations (•) in the (^13^C, ^15^N) isotope-filtered, ^15^N- or ^13^C-edited NOESY spectra from residues in molecule A to residues in molecule A’ Schematic representation of the secondary structure of the SAM_C_ domain is presented above and on the right side of the plot. Shading in the plot defines secondary structure regions.

The unique feature of the SLy1 SAM_C_ symmetric homodimer is the dimer interface, which has not been observed previously for SAM domains. This interface buries ~1,200 Å^2^ surface area. Most direct information on the interface can be derived from intermonomer proton-proton distances detected in isotope-filtered 3D NOESY experiments. The contact map in Fig. 3 provides insight into the architecture of the dimer interface. The majority of intermonomer contacts is formed between protons in the long helix α5 and the symmetrical partner helix α5′. Additional contacts are found between protons located in the C-terminal halves of helices α1 and α5′, respectively. The third group of short intermonomer contacts is found between protons in the C-termini of both monomers. In addition, NOE cross peaks were observed between the N-terminal P254 and the C-terminal end of helix α5′, and between the C-terminal Y316 in one monomer and L261 in helix α1′ as well as L281 in helix α3′ of the other monomer. In accordance with the intermonomer NOE data, the NMR structure shows that the SAM_c_ monomers face each other with the two α5 helices running in the same direction with additional contributions from α1 and the N-terminus (Fig. 2). The angle between the two helix axes of α5 and α5′ is ~−50°. The N-terminus and helix α1 are nearly perpendicular to helix α5 of the same monomer, and the side chains of residues in these three elements interact with side chains of the opposing helix α5′ in the other monomer.

**Figure 4:**
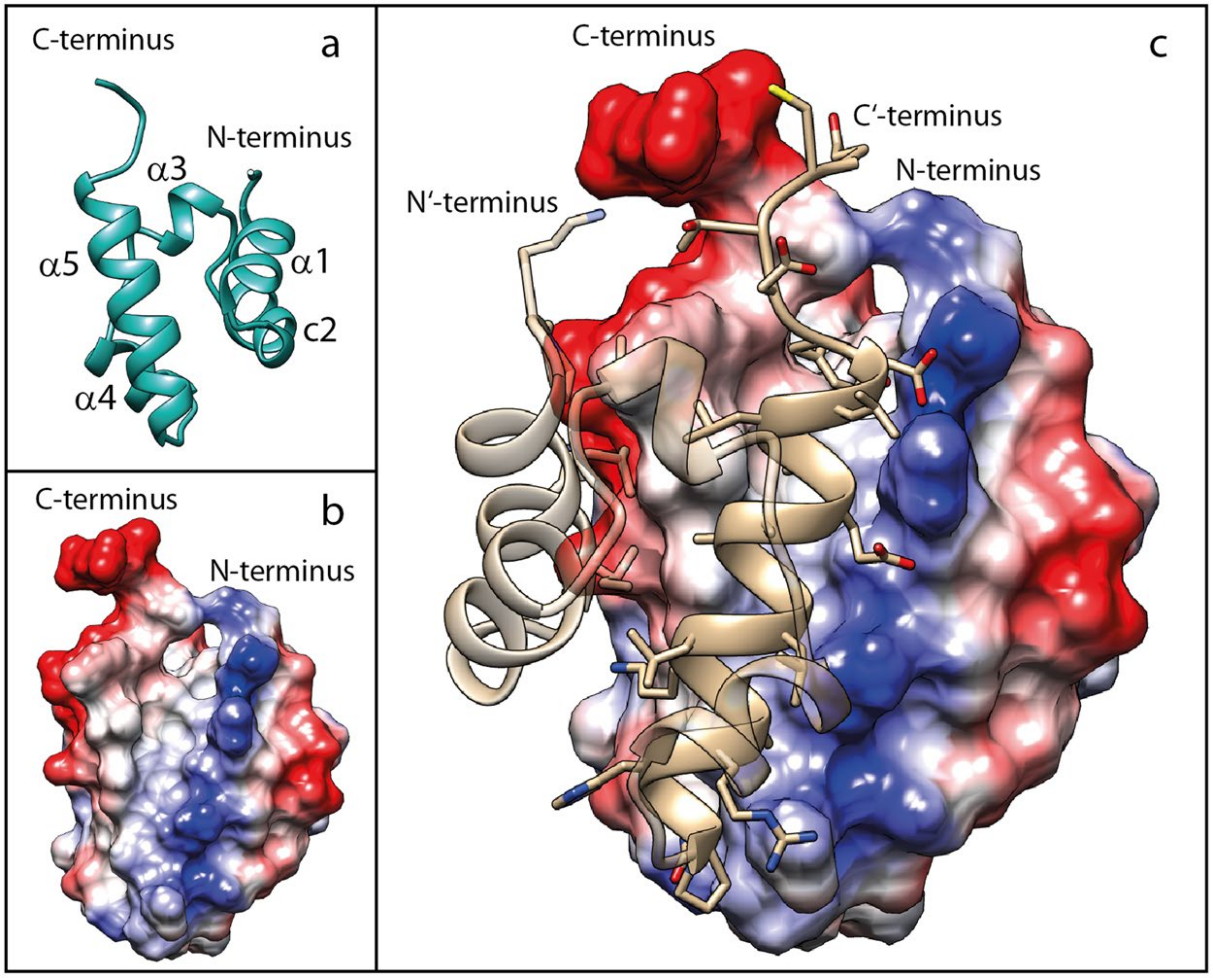
Surface complementarity of the SAM_C_ monomer. (**a**) Ribbon and (**b**) surface representation of the NMR structure of the SAM_C_ monomer are displayed in identical orientation. Surface colouring is based on electrostatic potential at pH 6.4 with negative charges in red and positive charges in blue. Helix α5 forms a hydrophobic ridge with negative charges on the left and hydrophobic residues on the right side. A positively charged ridge formed mainly by side chains of helices α1 and c2 exists. There is a hydrophobic groove between the two ridges. (**c**) Helix α5′ of the second monomer fits into this hydrophobic groove and side chains E311 and D315 of helix α5′ form salt bridges with residues that are part of the positively charged ridge.

**Figure 5:**
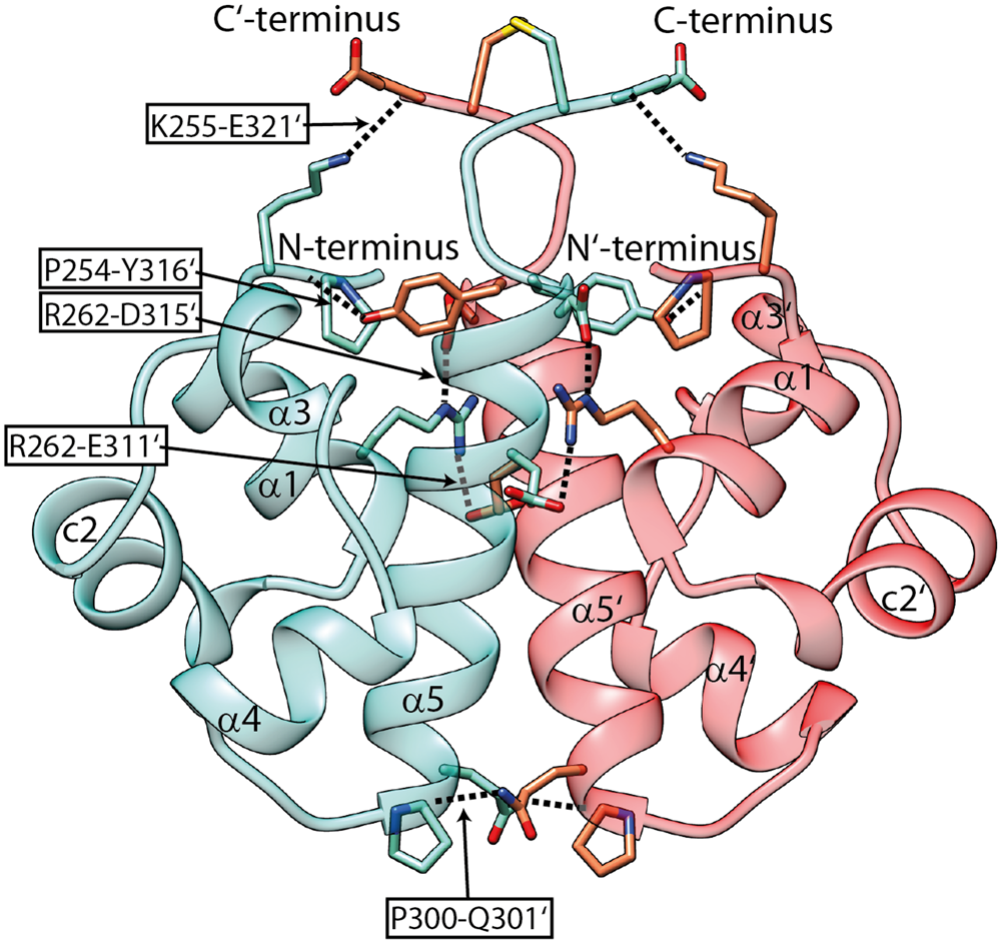
SLy1 SAM_C_ homodimer interface is stabilized by hydrogen bonds and salt bridges. H-bonds and salt bridges between the two monomers of the SAM_C_ homodimer shown in teal and red are labelled. Details on the stabilizing bonds are given in Supplementary Table S9. Side chains of interacting amino acids are shown in stick representation in the ribbon representation of the NMR structure.

The interacting surfaces of the two monomers display matching hydrophobic regions and charge complementarity in the SAM_C_ dimer (Fig. 4). Hydrophobic side chains of helix α5 extend into the hydrophobic groove formed by helices α1′, c2′ and α5′ of the other monomer. Analysis of the atomic coordinates of the dimer structure identified 12 potential intermonomer hydrogen bonds and salt bridges that stabilize the dimer (Fig. 5 & Supplementary Table S9). In particular, a network of such non-covalent interactions is suggested between residues in the N-terminus (P254, K255) or in helix α1 (R262) of one monomer with residues in the C′-terminus (Y316, E321) and in the C-terminal halve of helix α5′ (E311, D315) of the second monomer. A hydrogen bond between P300 of helix α5 and Q301 of helix α5′ is present and likely pulls the two helices together at their N-termini. The stabilizing disulphide bond between cysteines in position 320 adopts a negative right handed staple (−RHStaple) conformation^41^ in 14 out of the 15 analysed structures and a negative left handed staple (−LHStaple) conformation in one model.

### Crystal structure of SAM_wt_

In parallel, we determined the crystal structure of SAM_wt_ to a resolution of 2.05 Å. The structure belongs to the tetragonal space group P 4_1_ 2_1_ 2 with one molecule in the asymmetric unit. Details on data and refinement statistics are summarized in Supplementary Table S10. The final structure comprised 65 of the expected 69 residues (P254–D317 plus the N-terminal glycine). No electron density was visible for T318–E321 in the weighted 2Fo-Fc map, most likely because of conformational flexibility at the termini. The 3D-structure adopts the typical five helix bundle of a SAM domain with the following secondary structure elements: α1 (L257–R263), α3 (L281–F284), α4 (E289–E294) and α5 (P300–Y316). In addition, a composite helix c2 (E267–L274) was identified consisting of a 3_10_-helix (E267–H269) and an α-helical segment (T270–L274).

### The crystal structure of SAM_wt_ and the NMR structure of SAM_c_ share the same architecture

**Figure 6:**
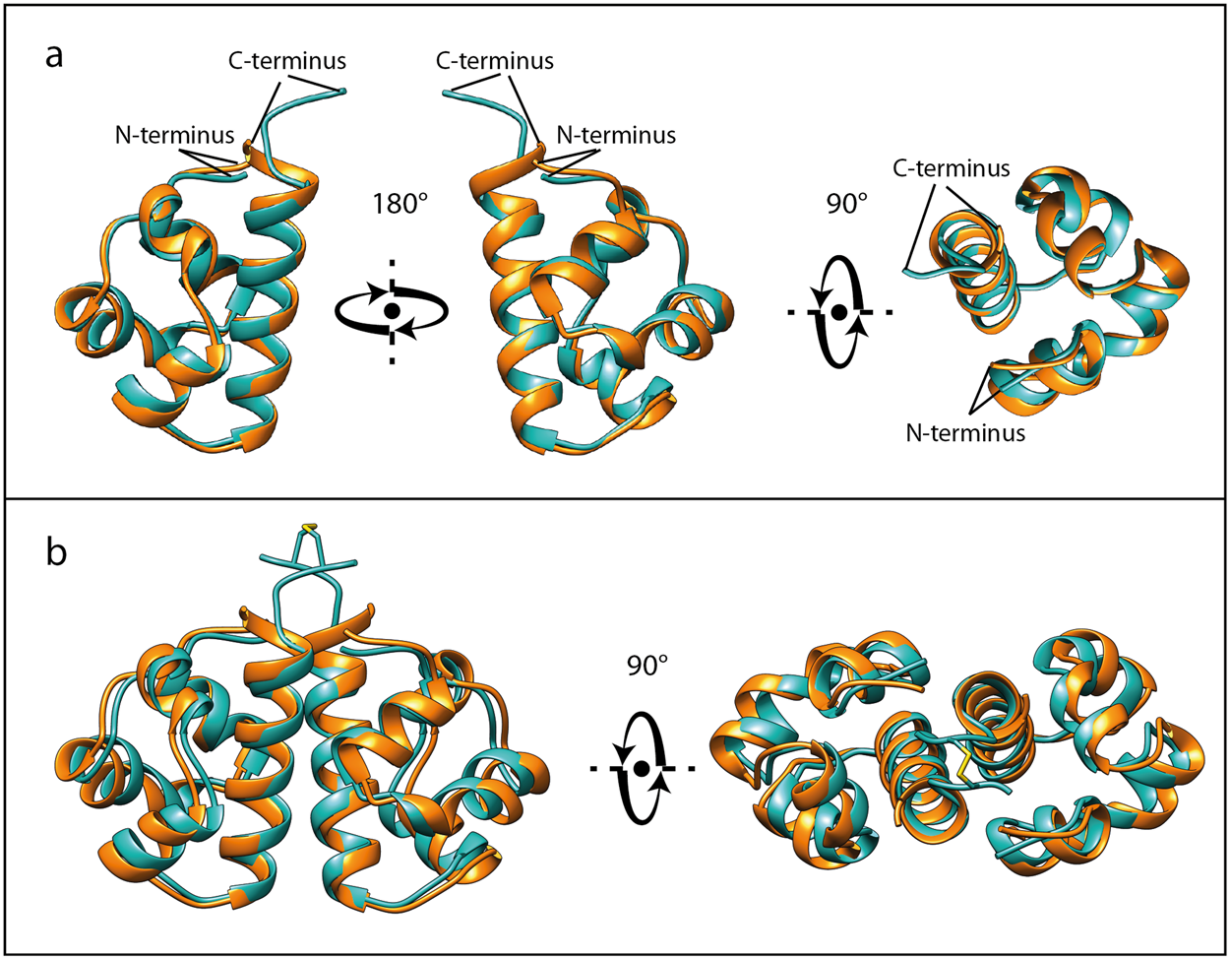
Overlay of SAM_C_ NMR and SAM_wt_ X-ray structures. The SAM_C_ structure closest to the average backbone structure of the ensemble of NMR structures of SAM_C_ (teal) and the X-ray structure of SAM_wt_ (orange) are depicted. (**a**) Superposition of SLy1 SAM monomers (C_α_ r.m.s.d. = 0.84 Å) is shown. (**b**) The superposition of the SAM_C_ homodimer with the crystallographic dimer of SAM_wt_ shows nearly identical structures with a C_α_ r.m.s.d. of 1.13 Å for the dimer.

The average NMR structure of the SAM_C_ monomer and the X-ray structure of SAM_wt_ are almost identical (Fig. 6a) with a C_α_ r.m.s.d. between the two monomers of 0.84 Å. The composite helix c2 is observed in both structures. The length of four helices differs slightly between the two structures: helices α1, c2, and α4 are one residue shorter in the X-ray structure (I264, L275, and L295, respectively) while α5 is one residue longer (up to Y316), according to DSSP analysis.

Although the asymmetric unit contained only a single molecule, inspection of symmetry related molecules in crystallographic complexes can reveal the presence of a multimeric state with sufficiently high dissociation free energies to form stable macromolecular assemblies. The PISA web server was used to calculate possible multimeric states, accessible/buried surface area, and the free energy of dissociation. The highest dissociation free energy was obtained for a crystallographic dimer with 2-fold crystallographic symmetry in which helices α5 and α5′ point in the same direction and are located in close proximity to each other. A free energy gain of 4.7 kcal mol^−1^ and a buried surface area of ~870 Å^2^ upon SAM_wt_ symmetric dimer formation were obtained. The lower buried surface area (difference of ~330 Å^2^) when compared with that of the NMR structure arises from the four residue shorter C-terminus in the SAM_wt_ crystal structure.

Hydrogen bonds and salt bridges between amino acids in helices α1 and α5′ and between P300 and Q301 at the N-terminus of helices α5 and α5′, respectively, stabilize the dimer interface. A list of all hydrogen bonds and salt bridges in the crystallographic SAM_wt_ dimer is presented in Supplementary Table S9. Equivalent H-bonds and salt bridges are seen in the NMR structure of SAM_C_. Superposition of the NMR structure of the SAM_C_ homodimer with the crystallographic SAM_wt_ homodimer confirms a high global identity with a C_α_ r.m.s.d. of 1.13 Å (calculated with LSQMAN^42^) (Fig. 6b).

### Extension of the N-terminus of SAM_wt_ increases the stability of the homodimer

**Figure 7:**
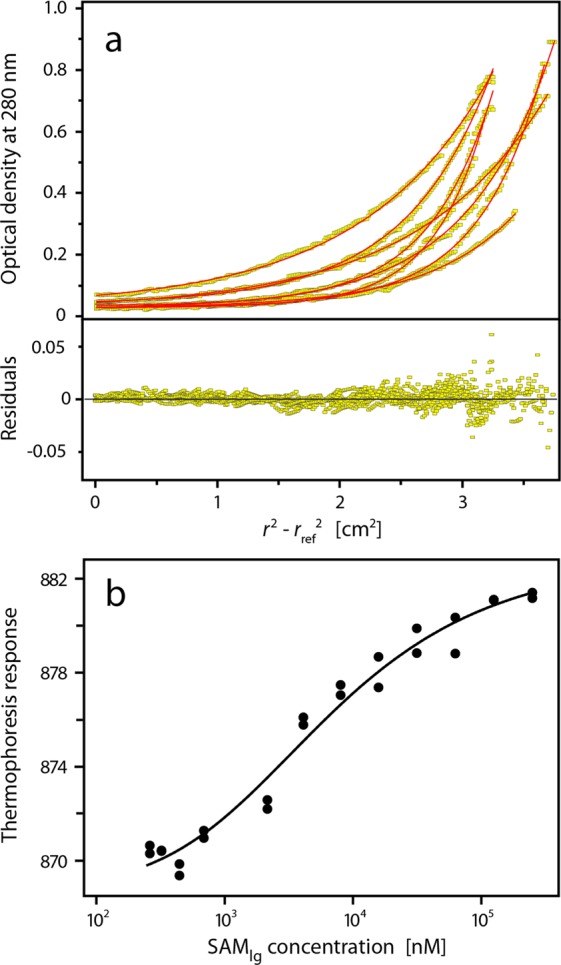
Analysis of the monomer-dimer equilibrium of SAM_lg_. (**a**) Sedimentation equilibrium experiments were performed using samples with different SAM_lg_ concentrations (31, 64, and 98 µM) at multiple speeds. Data were fit globally with a monomer-dimer model. The upper panel shows concentration profiles recorded after establishment of equilibrium between sedimentation and back diffusion and the calculated concentration distributions (red lines) based on a monomer-dimer equilibrium model. The global fit gives a *K*_d_ = 2.2 (1.8, 2.6) μM. The lower panel shows the residuals of the fit. (**b**) Binding isotherm from microscale thermophoresis data. The fit of the experimental data to a monomer-dimer equilibrium model (solid line) yields a *K*_d_ of (5.4 ± 1.4) μM.

The SAM_C_ structure presented in Fig. 4c shows a group of surface-exposed negatively charged residues (D317, E321) in the C-terminal area. The N- and C-termini of adjacent SAM_C_ monomers are in close spatial proximity (Figs 2c and 4c) and SLy1 has a high density of positive charges (K250, R251, K253, K255) in the sequence preceding the SAM core domain (Supplementary Fig. S2). Thus, we hypothesize that these residues form electrostatic interactions that further increase the affinity of SAM homodimerization. In order to test this, an N-terminally extended SLy1 SAM domain construct (SAM_lg_) was produced (Supplementary Fig. S2). Analytical ultracentrifugation (AUC) and MST experiments were used to examine the self-association of SAM_lg_. The analysis of the data revealed that the SAM_lg_ undergoes a monomer-dimer equilibrium with a stronger affinity reflected by the *K*_d_ values of 2.2 (1.8, 2.6) µM and (5.4 ± 1.4) µM obtained by AUC and MST, respectively (Fig. 7). Comparison with the dissociation data for SAM_wt_ (Supplementary Table S11) reveals that the extended SAM_lg_ shows an affinity increase by a factor of 30 to 50.

Translational diffusion coefficients (*D*) of SAM_lg_ particles at 30 °C were determined by NMR spectroscopy to complement the AUC and MST data. Linear regression of the diffusion coefficients measured at three concentrations (0.1, 0.5, and 1 mM) to zero concentration provided the diffusion coefficient at infinite dilution (*D*_0_). Based on the model of spherical particles, the hydrodynamic radius *R*_h_ with a value of (20.6 ± 0.5) Å was calculated. Wilkins *et al*. established an empirical relation *R*_h_ = 4.75 N^0.29^ Å between the hydrodynamic radius and the number of residues in the polypeptide chain, N, for natively folded proteins^43^. Applying this relation to SAM_lg_ (N = 75) predicts *R*_h_ = 16.6 Å for monomers and 20.3 Å for dimers. Thus, the diffusion data support the hypothesis of a predominantly dimeric oligomerization state of SAM_lg_ in the protein concentration range from 0.1 to 1 mM. This agrees well with the *K*_d_ results where over the concentration range of 0.1–1 mM the calculated fraction of SAM_lg_ in the dimer state is 85–95%.

## Discussion

In the present study, complementary biophysical and structural techniques were combined to characterise the self-association behaviour of the SLy1 SAM domain. SAM_wt_ exists in a monomer-dimer equilibrium with a *K*_d_ of 117 μM as determined by AUC, which was supported by MST results. Dimerization was also confirmed for SAM_lg_ by AUC, MST and NMR-based diffusion measurements.

Stabilization of the SAM_C_ homodimer by a disulphide bond outside the predicted core domain enabled structure determination by NMR spectroscopy. Similar strategies have been used successfully in NMR structural studies of homo-^32^ and heterodimers^44^ where disulphide bonds have been introduced to quench chemical exchange processes that have hampered NMR investigations. The introduction of the disulphide bond into the SAM homodimer yielded almost identical resonance positions in the 2D ^1^H-^15^N HSQC spectra of SAM_C_ in the reduced and oxidized state, indicating that the disulphide bond did not perturb the homodimer structure (Supplementary Fig. S6). Nonetheless, comparison of the 2D ^1^H-^15^N HSQC spectra of SAM_wt_ and disulphide-bonded SAM_C_ homodimer revealed larger than average chemical shift differences for some resonances from the N-terminal region (K255, T256), the second half of α-helix 5 (L313, L314, D315) and from C-terminal residues close to the S320C mutation site (Supplementary Fig. S7). These observed chemical shift differences arise (besides the local differences caused by the S320C exchange) predominantly from the SAM_wt_ existing in the monomer-dimer equilibrium, where the population of monomer is ~17% at the NMR sample concentration of 1.4 mM. Therefore, the chemical shift of resonances in the 2D ^1^H-^15^N HSQC spectrum of SAM_wt_ is affected by this monomer population. In addition, the reduced mobility of the C-terminus of cross-linked SAM_C_ will limit sampling of conformational space and thus influence the chemical shift of resonances associated with these terminal residues.

**Figure 8:**
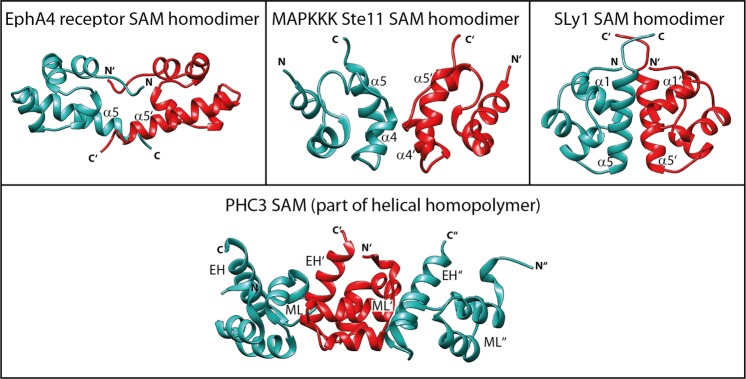
SAM domain interfaces in homodimers and homopolymers. Identical surfaces of two SAM monomers form the interface of symmetric SAM homodimers. The upper row shows the three types of SAM homodimers that have been reported. The termini-mediated EphA4 receptor SAM homodimer (PDB: 1B0X)^16^, the MAPKKK Ste11 SAM homodimer stabilized by interactions between amino acid residues in helices α4 and α5 (PDB: 1X9X)^17^, and the SLy1 SAM homodimer stabilized by interactions between amino acid residues in helices α1, α5 and the termini of both monomers (PDB: 6G8O). The monomer-monomer interface in a SAM homopolymer is formed by two different surfaces: the mid-loop (ML) surface of one monomer and the end-helix (EH) surface of the other monomer. This feature enables oligomerization. Three PHC3 SAM monomers that belong to a left-handed helical structure with six monomers per turn (PDB: 4PZO)^75^ are displayed in the lower panel.

The structure of the SLy1 SAM domain dimer was solved by NMR spectroscopy and X-ray crystallography. Since only one SAM_wt_ molecule was found in the asymmetric unit of the crystal structure possible SAM assemblies and interfaces were determined from the analysis of the symmetric equivalent SAM_wt_ molecules using PISA. The only energetically favourable SAM_wt_ dimer interface is in agreement with the intermonomer NOE data recorded. The crystallographic homodimer closely matches the SAM_C_ solution structure with a low C_α_ r.m.s.d. of 1.13 Å. In both cases, the two SLy1 SAM domains predominantly interact through their helices α5 with the long sides of the helices packing against each other, and additional contributions from helix α1, c2 and the N-terminal residues (Fig. 6b). Future mutational studies can build on our structural data and should help to demarcate the role of key residues required for SLy1 SAM homodimerization. SAM domains frequently self-associate; however, symmetric homodimerization of SAM domains is uncommon. Currently, structures of only two SAM homodimers have been deposited in the PDB^16,17^, whereas other forms of SAM domain homo- and heterooligomers are mediated by the asymmetric EH-ML interface (cf. Fig. 8, lower panel). In both reported SAM homodimers, the dimer interface differs from the interface observed in the SLy1 SAM homodimer (Fig. 8). A symmetric homodimer was observed in the crystal structure of the isolated EphA4 SAM domain (PDB ID: 1B0X)^16^. N- and C-terminal ends of the EphA4 SAM monomer structure including the C-terminal part of the long helix α5 point away from the core structure of the SAM domain and interdigitate with the termini of the other monomer (Fig. 8). The antiparallel termini represent the major interface between the EphA4 SAM monomers with additional interactions from side chains of helices α1 and α3^16^. The other reported SAM homodimer was determined by NMR spectroscopy for the SAM domain of the yeast protein Ste11 (PDB ID: 1X9X)^17^. The interface between the Ste11 SAM monomers is formed by the N-terminal half of helix α5 that is packed against the parallel running helix α4′ of the other monomer (Fig. 8). These two SAM homodimers show low stability in solution, as indicated by dissociation constants in the single digit millimolar range (Supplementary Table S11)^16,18^, which is ~4 to 40-fold weaker than the corresponding affinity measured for the SLy1 SAM_wt_ homodimer. Inspection of the three SAM homodimer structures reveals that the buried surface area of the SLy1 SAM_wt_ homodimer (870 Å^2^) is larger than that of the Ste11 homodimer (504 Å^2^) but moderately lower than that of the EphA4 SAM homodimer (1,009 Å^2^). Although the EphA4 SAM homodimer has a buried surface area of similar size to that of the SLy1 SAM homodimer, the number of stabilizing hydrogen bonds and salt-bridges is noticeably lower (2 versus 12), which likely explains the observed difference in homodimer stability. In contrast, for the Ste11 homodimer a similar number of hydrogen bonds and salt bridges are present (8), but the buried surface area is considerably smaller and thus the homodimer is less stable.

The close proximity of the N- and C-termini of SAM_C_ coupled with their complementing charges (i.e., D317 and E321 at the C-terminus and K250, R251, K253 and K255 at the N-terminus) suggests further dimer stabilizing interactions between SLy1 proteins involving residues that are not part of the SAM domain. This is supported by the observed strong influence of SLy1 residues G249−K253 on dimer stability (Fig. 7). Extension of the SAM_wt_ N-terminus by these five residues increased the affinity of the SAM domain dimerization by a factor of 30−50. The *K*_d_ of SAM_lg_ is therefore comparable to the *K*_d_ observed for SAM domain homo- and hetero-associations, for which biological relevance has been ascertained (e.g., Yan SAM fibrils^22^ or the heterodimers of Odin SAM1/Arap3 SAM^25^, Odin SAM1/EphA2 SAM^26^ and Ship2 SAM/EphA2 SAM^27^), suggesting that the SLy1 SAM dimerization is of functional significance for SLy1. Self-association is not uncommon for adapter proteins and proteins involved in cell signalling. 14–3–3 proteins have been identified as binding partners of SLy1^5^. Interestingly, 14-3-3 proteins function as dimers and have been shown to interact with other dimeric proteins. Thus, SLy1 may interact as a dimer with 14-3-3 proteins.

Besides the SAM domain of Ste11 forming a homodimer, this domain also interacts with the SAM domain of the Ste11 regulator Ste50 through its ML surface, forming a distinct heterodimer through a ML-EH interaction. Since the structure of the SLy1 SAM homodimer contains an exposed ML surface on each monomer, it is plausible that other SAM domain-containing proteins could interact with SLy1 SAM in its dimerized state via an ML-EH interaction, thereby enabling SLy1 to mediate interactions between other SAM domain-containing proteins. Consequently, modulation of the SLy1 SAM domain dimerization would affect the type of protein-protein interactions mediated by SLy1. Two putative phosphorylation sites in SLy1, T318 and S320, are in close proximity to the SAM homodimer interface^45^. Currently, no biological function has been assigned to these phosphorylation sites. However, phosphorylation at either of the two residues would likely affect SAM dimerization kinetics and regulate dimerization, as has been shown for interface proximal phosphorylation sites in other proteins that dimerize^46,47^. Characterising the role phosphorylation has in modulating SAM dimer affinity and identifying binding partners to SLy1 SAM should further our understanding of the SLy1 interactome.

In conclusion, the SAM domain of the adapter protein SLy1 homodimerises through a novel interaction interface. This homodimer is significantly more stable than other reported SAM homodimers, especially in the presence of an extended N-terminus, suggesting that SLy1 functions as a dimer. Such dimerization provides a larger interaction surface for SLy1 to function as an adapter protein.

## Materials and Methods

### Cloning and recombinant protein production

The SAM domain of murine SLy1 (UniProtKB: Q8K352) comprises amino acid residues 254–316. A SLy1 SAM coding DNA fragment was cloned into a modified pGEX-6P-2 vector (GE Healthcare) with a unique Bsp120I restriction site using Bsp120I and XhoI. The complete gene product contains an N-terminal GST tag followed by a PreScission protease recognition sequence and residues 254 to 321 of SLy1. A longer version of the described plasmid contains DNA coding for residues 249 to 321 of SLy1. A third plasmid encodes residues 254–321 of SLy1 but with cysteine instead of serine at position 320 (Supplementary Fig. S2).

The fusion proteins were expressed in LB medium with 100 µg/mL ampicillin using *Escherichia coli* BL21(DE3) cells transformed with one of the prepared plasmids. Cells were harvested by centrifugation and stored at −80 °C. Cells were resuspended in lysis buffer (PBS, 100 µg/mL lysozyme, 20 µg/mL DNAse A, EDTA-free protease inhibitor cocktail Complete (Roche)), incubated at room temperature with gentle mixing for 1 h and mechanically disintegrated (Branson 250 rod sonifier (Branson Ultrasonics, Danbury, CT, USA) or microfluidizer M-110P (Microfluidics, Westwood, MA, USA)). Cell debris were pelleted by centrifugation and the supernatant was applied to a Glutathione Sepharose affinity column, washed with 2 × 5 CV GST binding buffer (PBS, 5 mM DTT, pH 7.4) and 2 × 5 CV PreScission cleavage buffer (50 mM Tris, 150 mM NaCl, 5 mM DTT, pH 7). The slurry was incubated with PreScission protease (GE Healthcare) at 10 °C under gentle agitation overnight. The SAM containing flow-through was concentrated using an Amicon stirred cell (50 mL, MWCO 3000), passed through a 0.2 µm syringe filter and fractionated on a size exclusion column (HiLoad 26/60 Superdex 75 pg, GE Healthcare). The running buffer was 50 mM potassium phosphate, 20 mM NaCl, pH 6.4. Cysteine-containing SAM was purified under reducing conditions. Amino acid sequences of the three produced SLy1 SAM variants SAM_wt_, SAM_C_, and SAM_lg_ are listed in Supplementary Fig. S2. Expression of uniformly labelled [U-^15^N] or [U-^13^C, ^15^N] protein was carried out in M9 minimal medium containing (^15^NH_4_)SO_4_ or both (^15^NH_4_)SO_4_ and ^13^C-glucose, respectively. Purity of the SAM constructs was confirmed by SDS PAGE. Biophysical studies were conducted in standard buffer (50 mM potassium phosphate, 20 mM NaCl, 0.1 mM EDTA, 0.03 wt% NaN_3_, pH 6.4) except for NMR experiments, where the standard buffer was supplemented with 7% ^2^H_2_O (referred to as NMR buffer).

### Analytical ultracentrifugation

Sedimentation equilibrium experiments were performed with Beckman Optima XL-A (SAM_lg_) and ProteomLab X-LA (SAM_wt_) ultracentrifuges (Beckman-Coulter, Brea, CA, USA) equipped with absorption optics. Protein samples (120 µl each) in standard buffer without NaN_3_ were loaded into standard aluminium double sector cells with quartz windows and an optical path length of 12 mm. The reference sector was filled with buffer. Centrifugation was performed at a temperature of 30 °C and different speeds using An-50 Ti Analytical 8-Place (SAM_wt_: 28,000; 35,300; and 42,700 rpm) and An-60 Ti Analytical 4-Place (SAM_lg_: 31,900; 39,000; 45,100; and 50,400 rpm) titanium rotors (Beckman-Coulter). Three samples with different loading concentrations (SAM_wt_: 60, 120, and 300 µM; SAM_lg_: 31, 64, and 98 µM) were analysed for each SAM variant. Sedimentation equilibrium profiles were recorded at 280 nm in radial step mode with a 10 µm (30 µm) step size and 20-point (5-point) averaging for SAM_wt_ (SAM_lg_). Data evaluation was carried out using the global equilibrium fitting module of UltraScan II software (ver. 9.9) (http://www.ultrascan.uthscsa.edu). All sedimentation equilibrium profiles with sufficiently high information content were used in the global fits. Parameters required for data analysis were derived using the following tools (protein specific parameters are based on amino acid sequence): partial specific volume of the proteins and the mass density of the buffer were calculated with SEDNTERP (ver. 20120828 BETA; http://www.jphilo.mailway.com/download.htm#SEDNTERP). Monomer molecular mass and the molar extinction coefficient at 280 nm were estimated with ProtParam (http://expasy.org/tools/protparam.html). Absorbance profiles were converted into molar concentration profiles prior to global nonlinear least-squares fits of the equilibrium data to different interaction models. Three models were tested: (i) monomer-dimer equilibrium of reversibly self-associating species; (ii) single ideal species (one-component model); (iii) two ideal, non-interacting species (two-component model).

### Microscale thermophoresis

A Monolith NT.015 device (NanoTemper Technologies GmbH, Munich, Germany) was used^48^. SAM_wt_ and SAM_lg_ were fluorescent labelled with the amine-reactive Alexa Fluor 488 dye (NT labelling kit BLUE). Dilution series of the proteins were prepared in standard buffer. The concentration of fluorescence labelled SAM_wt_ (70 nM) or SAM_lg_ (60 nM) and the total sample volume (10 μL) was kept constant within a dilution series. The total concentration of SAM_wt_ was varied between 1.1 and 824 µM (SAM_lg_: 0.2 and 250 μM). For each sample, 5 μL of protein solution was transferred into a NT Premium Coated Capillary (SAM_wt_) or NT Standard Treated Capillary (SAM_lg_). The binding isotherm was recorded at room temperature. Experimental data were fitted to a monomer-dimer equilibrium.

### NMR experiments

For structure determination, 1.4 mM [U-^15^N, ^13^C] or [U-^15^N] labelled SAM_C_ in NMR buffer was used at 35 °C, unless otherwise noted. Data were recorded on Varian Unity Inova or VNMRS and Bruker Avance III HD NMR spectrometers equipped with cryogenically cooled z-gradient probes operating at ^1^H frequencies of 600, 700 and 900 MHz. Backbone, and aliphatic and aromatic side chain ^1^H, ^15^N and ^13^C resonance assignments for the SAM_C_ homodimer were obtained from multidimensional heteronuclear NMR experiments. Proton chemical shifts were referenced to 2, 2-dimethyl-2-silapentane-5-sulfonate (DSS), whereas the ^15^N and ^13^C chemical shifts were indirectly referenced according to the ratios given by Wishart *et al*.^49^. Data sets were processed using NMRPipe^50^ and analysed by CcpNMR Analysis^51^.

### NMR-based structure calculations

Interproton distance restraints were derived from 3D NOESY-[^1^H-^15^N]-HSQC (mixing time *τ*_mix_ = 150 ms), NOESY-[^1^H-^13^C]-HSQC (aliphatic region, τ_mix_ = 120 ms), NOESY-[^1^H-^13^C]-HSQC (aromatic region, *τ*_mix_ = 140 ms) experiments on the [U-^13^C,^15^N] SAM_C_ dimer. Intermonomer ^1^H-^1^H distances were provided by 3D ω1-(^15^N, ^13^C)-filtered NOESY-[^1^H-^15^N]-HSQC (*τ*_mix_ = 200 ms) and ω1-(^15^N, ^13^C)-filtered NOESY-[^1^H-^13^C]-HSQC (*τ*_mix_ = 150 ms)^52^. Samples for isotope-filtered experiments were prepared by mixing equimolar amounts of reduced isotope-labelled and unlabelled SAM_C_. Subsequently, the sample was placed under oxidizing conditions to facilitate disulphide bond formation. The final concentration of labelled protein in this sample was 0.8 mM.

Backbone ϕ and ψ angles were derived from experimental ^13^C_α_, ^13^C_β_, ^13^C′, ^15^N^H^, ^1^H^N^, ^1^H_α_ chemical shifts using TALOS+^34^. Side chain χ_1_ torsion angles were deduced from experimental ^3^*J* coupling constants based on the empirical Karplus relation for χ_1_ using self-consistent Karplus parameters^53^. Quantitative ^3^*J*_NHβ_-HNHB and ^3^*J*_HαHβ_-HAHB(CACO)NH experiments were recorded to measure ^3^*J* couplings. Interresidue N_i_-H^N^ ∙∙∙∙ O = C_j_′ hydrogen bonds were detected by the observation of ^h3^*J*_NC′_ couplings in a 2D long-range HNCO experiment^54^.

Version 2.3.2 of ARIA (Ambiguous Restraints for Iterative Assignment) was used for NOESY cross peak assignment and structure calculation^35,36^. NOE cross peak assignments of the acquired NOESY spectra were obtained by an iterative procedure using a combination of manual and automatic steps. The tolerances for automatic assignments by ARIA were 0.04–0.05 and 0.04–0.06 ppm for the ^1^H direct and indirect dimensions, respectively, and 0.5 ppm for the heteronuclear dimensions. Experimentally detected H-bonds, experimental χ_1_ and TALOS derived ϕ and ψ torsion angles as structure restraints were also used. A homodimeric SAM_C_ structure with C2 symmetry was explicitly assumed and the non-crystallographic symmetry option was active^31,33^. The disulphide bridge between C320 and C320′ was introduced as a covalent bond. Structures were calculated by a combination of ARIA and CNS v1.2142^55^ (including the ARIA patchset) using the PARALLHDG force field with a log-harmonic potential^56^ and automatic restraint weighting. In the final iteration, 100 structures were calculated and refined in explicit water. The 15 lowest-energy SAM_C_ structures were selected for further analysis.

The stereochemical quality of the refined models was assessed in PROCHECK NMR^57^. Agreement of the NOE data with the calculated structures was assessed using the RPF tool (http://nmr.cabm.rutgers.edu/rpf)^37^. Assignment of secondary structure elements (SSE) based on protein coordinates was performed by DSSP^39,40^. The webserver PDBeFold (http://www.ebi.ac.uk/msd-srv/ssm/) was used for secondary structure based comparison and 3D alignment of SAM structures, referred to as secondary structure matching (SSM)^58^. The PISA webserver (http://www.ebi.ac.uk/pdbe/pisa/) was used to analyse the SAM_C_ homodimer and to derive potential contact-dependent and electrostatic interactions between the monomers from the atomic coordinates^59^.

### X-ray crystallography

Purified SAM_wt_ (~10 mg mL^−1^) was transferred into Tris buffer (50 mM Tris-HCl, 150 mM NaCl, pH 7.0), sterilized by membrane filtration (0.2 µm) and used for crystallization by the vapour diffusion method. Crystals were grown in 1.4 µL sitting drops (0.7 µL protein solution and 0.7 µL reservoir solution) against a 70 µL reservoir (0.1 M K_2_HPO_4_, 2.2 M (NH_4_)_2_SO_4_, 0.1 M NaCl, 0.1 M imidazole). Crystals appeared after 5 to 7 days incubation at 19 °C. Crystals were cryoprotected by addition of 10% (v/v) glycerol, mounted in a fibre loop, flash cooled in a stream of cold (~100 K) nitrogen gas and stored in liquid nitrogen until use. X-ray diffraction data at 100 K were recorded at the beamline ID30A-3 of the European Synchrotron Radiation Facility (ESRF) in Grenoble, France, using an Eiger X 4 M detector (DECTRIS, Baden-Dättwil, Switzerland).

Diffraction images were processed with the program XDS^60^ resulting in integrated intensities for all diffraction spots. The program POINTLESS^61^ was used for space group identification. Scaling of the diffraction images and averaging of symmetry-related reflections was conducted with the program AIMLESS^62^. The number of molecules in the crystallographic asymmetric unit was determined using the Matthews coefficient^63^ provided by the CCP4 software suite^64^. The set of merged structure-factor amplitudes was then subjected to phasing. Initial phases were calculated with REFMAC5^65^ of CCP4 based on a structural model of SAM_wt_ generated by homology modelling on the SWISS-MODEL web server (https://swissmodel.expasy.org/)^66,67^ followed by molecular replacement with the program MOLREP^68^ in CCP4. The SAM domain of SAMSN1 (PDB ID: 1V38) was used for homology modelling. Model refinement was conducted with REFMAC5 and the program Phenix^69^. The molecular graphics software Coot^70^ was employed for visual inspection, building and manual improvement of the structural model. Crystallographic R-factors *R*_work_ and *R*_free_ were used to monitor the progress of the refinement process. The quality of the structure was validated with MolProbity^71^. Data collection and refinement statistics are listed in Supplementary Table S10.

Figures of structures were generated with UCSF Chimera^72^ using secondary structure assignments from the DSSP program^39^. The PISA webserver^59^ was used to analyse the multimeric state.

### Translational diffusion measurements

One-dimensional ^15^N-edited diffusion-ordered NMR spectroscopy (DOSY)^73^ was used to measure *D* of SAM_lg_. DOSY experiments were conducted at 30 °C on ^15^N-labelled SAM_lg_ at 0.1, 0.5, and 1 mM in NMR buffer. Extrapolation of the functional dependency of *D* on protein concentration to infinite dilution provides *D*_0_. Assuming a spherical shape of the diffusing particles, the simplified Stokes-Einstein relation was used to relate *D*_0_ to the hydrodynamic radius *R*_h_:

$${R}_{{\rm{h}}}=\mathrm{kT}/6\pi \eta {D}_{0}$$

The viscosity *η* of the solvent (90% H_2_O/10% ^2^H_2_O) was calculated as described previously^74^.

### Accession codes

NMR resonance assignments of the SLy1 SAM_C_ have been deposited in the BioMagResBank (accession code 27432). The NMR structures of the SLy1 SAM_C_ homodimer have been deposited in the Protein Data Bank (PDB ID: 6G8O). Atomic coordinates and structure factors for SLy1 SAM_wt_ have been deposited in the PDB (ID: 6FXF). The datasets generated and/or analysed during the current study are available from the corresponding author on reasonable request.

## Supplementary information

Supplementary Information
