# Supplementary material for: Structure of the SLy1 SAM homodimer reveals a new interface for SAM domain self-association
Source: Sci Rep. 2019 Jan 10;9:54. doi: 10.1038/s41598-018-37185-3 (PMC6328559; doi:10.1038/s41598-018-37185-3)
Supplement: Supplementary file 1 — Supplementary Information [file 41598_2018_37185_MOESM1_ESM.pdf]

Supplementary Information

Structure of the SLy1 SAM homodimer reveals a new interface for SAM domain self-association

Laura Kukuk[1,2], Andrew J. Dingley[1,2], Joachim Granzin[1], Luitgard Nagel-Steger[1,2], Pallavi Thiagarajan-Rosenkranz[1,2], Daniel Ciupka[2], Karen Hänel[1], Renu Batra-Safferling[1], Victor Pacheco[1,3], Matthias Stoldt[2], Klaus Pfeffer[4], Sandra Beer-Hammer[4,5], Dieter Willbold[1,2,]* & Bernd W. Koenig[1,2,]*

[1] Institute of Complex Systems, Strukturbiochemie (ICS-6), Forschungszentrum Jülich, 52425 Jülich, Germany

[2] Institut für Physikalische Biologie, Heinrich-Heine-Universität Düsseldorf, Universitätsstraße 1, 40225 Düsseldorf, Germany

[3] Institut für Makromolekulare Chemie, Albert-Ludwigs-Universität Freiburg, Stefan-Meier-Straße 31, 79104 Freiburg, Germany

[4] Institut für Medizinische Mikrobiologie und Krankenhaushygiene, Heinrich-Heine-Universität Düsseldorf, Universitätsstraße 1, 40225 Düsseldorf, Germany

[5] Institut für Experimentelle und Klinische Pharmakologie und Toxikologie, und Interfakultäres Zentrum für Pharmakogenomik und Arzneimittelforschung (ICePhA), Eberhard-Karls-Universität Tübingen, Wilhelmstraße 56, 72074 Tübingen, Germany

* Correspondence and requests for materials should be addressed to B.W.K. (email: b.koenig@fz-juelich.de) or D.W. (email: d.willbold@fz-juelich.de)

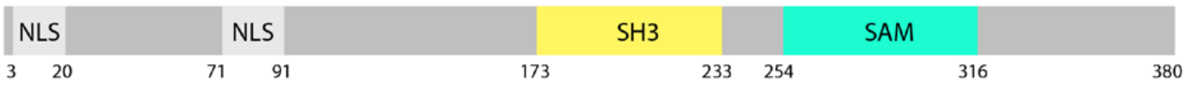

Supplementary Figure S1. Domain organization of the SH3 domain containing protein expressed in lymphocytes (SLy1). Murine SLy1 (UniProtKB: Q8K352) is 380 amino acids in length and the domain architecture is composed of a bipartite nuclear localization site (NLS), a Src homology 3 (SH3) domain and a sterile alpha motif (SAM) domain.

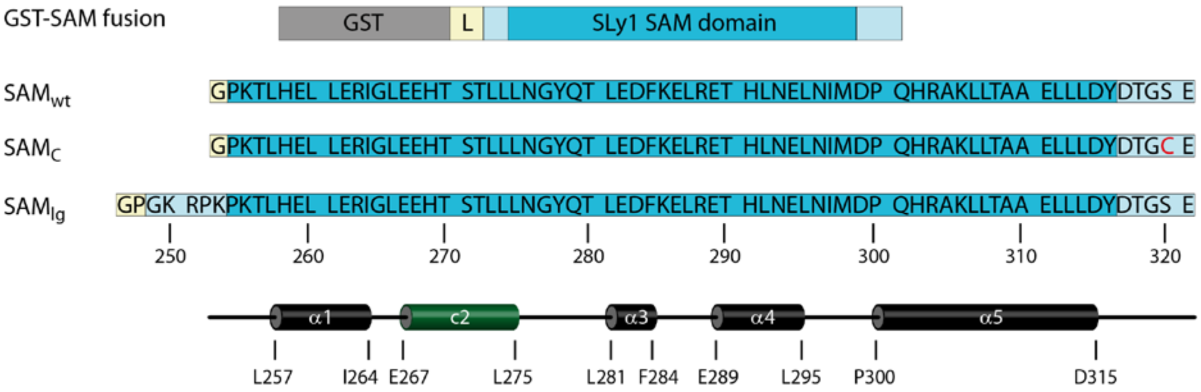

Supplementary Figure S2. Amino acid sequences of the SLy1 SAM domain constructs studied. A schematic representation of the fusion protein used for recombinant production of the SAM domain variants in *E. coli* is shown at the top. An eight residue linker (L, yellow) representing the PreScission protease recognition site connects glutathione S-transferase (GST, grey) with the SAM domain (blue) plus a few flanking amino acids from the sequence of SLy1 (light blue). The N-terminal glycine or glycine-proline dipeptide (yellow) is not present in SLy1, but is retained following enzymatic cleavage of the fusion protein. SAM_{wt} consisting of the SAM domain of SLy1 and five extra residues at the C-terminus was studied. SAM_C contains the S320C mutation (red). In addition, an N-terminally extended variant (SAM_{Ig}) was also used in this study. DSSP analysis of the NMR structure of SAM_C reveals five helices (below). Helix c2 (green) is split into a 3_{10}-helix (E267–H269) and an α-helix (T270–L275), whereas the other four are α-helices (black).

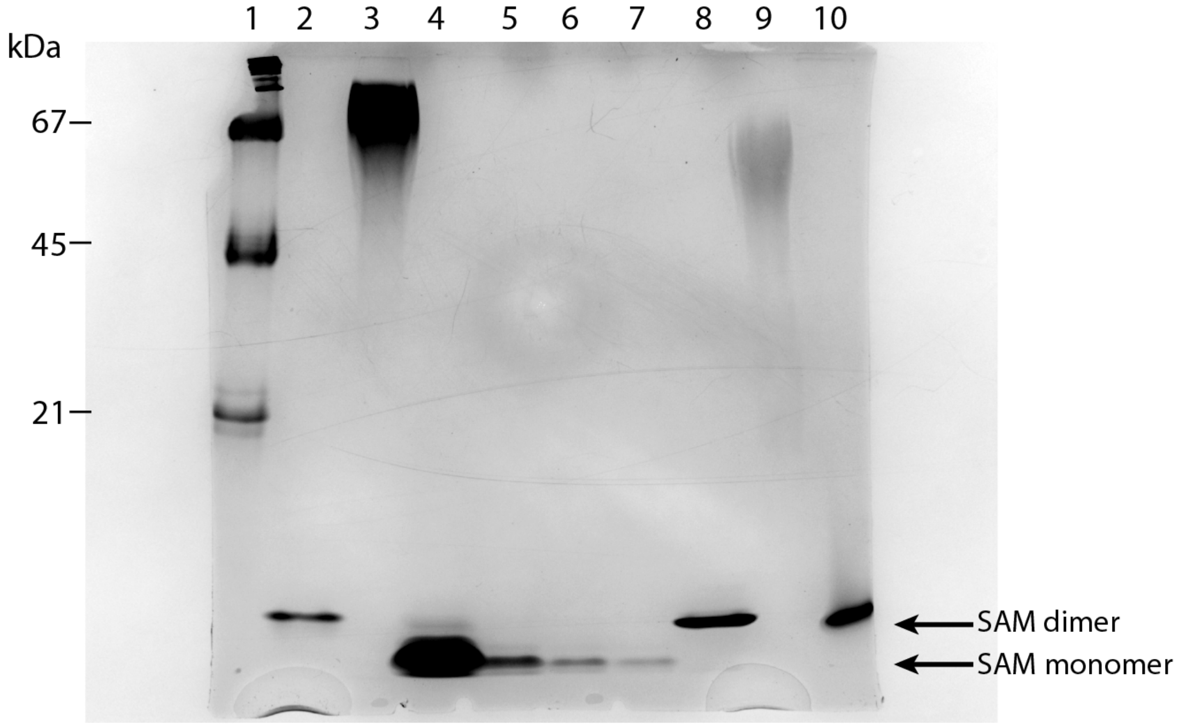

Supplementary Figure S3. Blue native PAGE reflects monomer-dimer equilibrium of SAM_{wt}. A concentration series of SAM_{wt} was analyzed on a homogeneous 20% acrylamide gel. Cross-linked SAM_C homodimer served as a control. (Lane 1: size standard (SERVA Native Marker, Liquid Mix for BN/CN); lane 4: 210 µM SAM_{wt}; lane 5: 26 µM SAM_{wt}; lane 6: 13 µM SAM_{wt}; lane 7: 6.5 µM SAM_{wt}; lane 8: 45 µM cross-linked SAM_C; lanes 2, 3, 9 & 10: unrelated proteins). SAM_{wt} homodimer is observed only at the highest loading concentration (210 µM) in agreement with the dimer dissociation constant determined by analytical ultracentrifugation and microscale thermophoresis.

The gel picture was recorded with a Gel Doc XR+ Imaging system (BioRad) using the software Image Lab (version 5.2.1). Standard settings for Coomassie Brilliant Blue stained protein gels were used with the exposure time automatically determined by the software using the option for intense bands. The digital image was exported in tagged image file format (tiff) and transferred to Adobe Illustrator CS5 (version 15.0.0) for annotation. The displayed image was not cropped or manipulated.

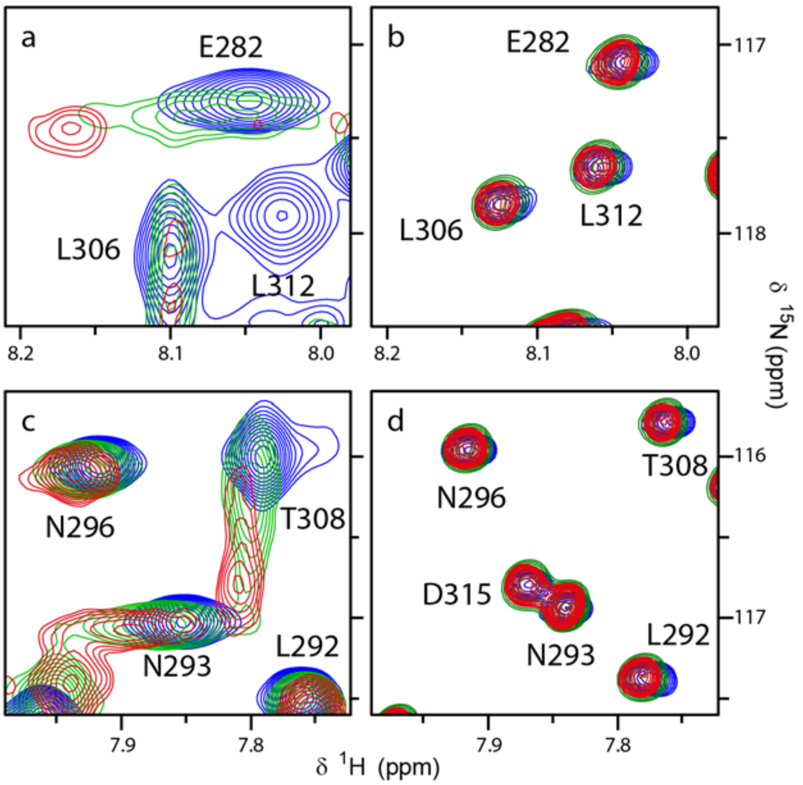

Supplementary Figure S4. Exchange broadening of SAM_{wt} resonances. Identical regions from 2D ^{1}H-^{15}N-HSQC spectra of SAM_{wt} (**a**, **c**) and cross-linked SAM_{C} (**b**, **d**) are shown. Overlay of spectra recorded at 14 (red), 50 (green) and 500 µM (blue) of SAM_{wt} displays concentration-dependent line broadening, variation in resonance intensities and chemical shift changes because of chemical exchange on the fast-to-intermediate time scale. Peak positions and line width show no concentration-dependent effects for the disulphide bond-stabilized SAM_{C} (red: 14 µM; green: 140 µM; blue: 1,400 µM) homodimer. Formation of a disulphide bond between the cysteines at position 320 of two SAM_{C} molecules quenches the chemical exchange process.

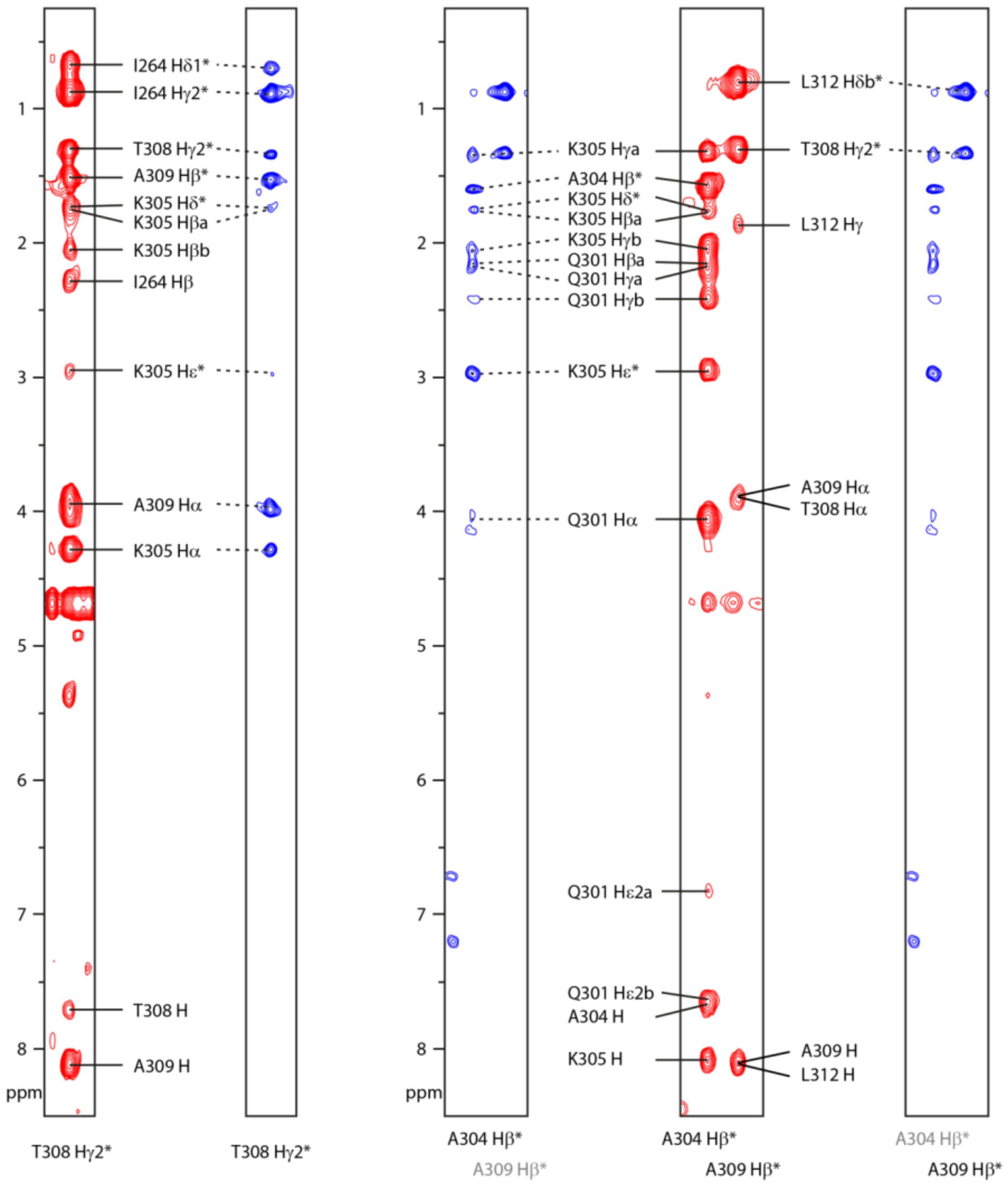

Supplementary Figure S5. Representative strips from isotope-filtered NOESY spectra recorded for the disulphide bond-stabilized SAM$_C$ dimer and SAM$_{wt}$. Exemplary strips from identical regions of the 3D and 2D ^{13}C/^{15}N-filtered, ^{13}C-edited NOESY spectra of SAM$_C$ (red) and SAM$_{wt}$ (blue), respectively, are presented. Spectra were recorded at ^{1}H frequencies of 700 MHz (SAM$_C$; 32 scans) and 900 MHz (SAM$_{wt}$; 768 scans) at 35 °C. Intermolecular cross correlations of T308 Hγ2* protons and the Hβ* protons of A304 and A309 with protons of the adjacent monomer are observed. Intermonomer cross peaks for SAM$_C$ are labelled with assignment information. All intermolecular cross correlations observed for SAM$_{wt}$ have matching peaks in the ^{13}C/^{15}N-filtered NOESY of SAM$_C$ (dotted lines). These observations show that the interface of the SAM homodimer is unaltered in the disulphide bond-stabilized SAM$_C$ homodimer. Chemical exchange-induced line broadening is responsible for the much weaker (or missing) intensity of the cross correlations in the spectrum recorded on SAM$_{wt}$. This chemical exchange arises from the monomer-dimer equilibrium.

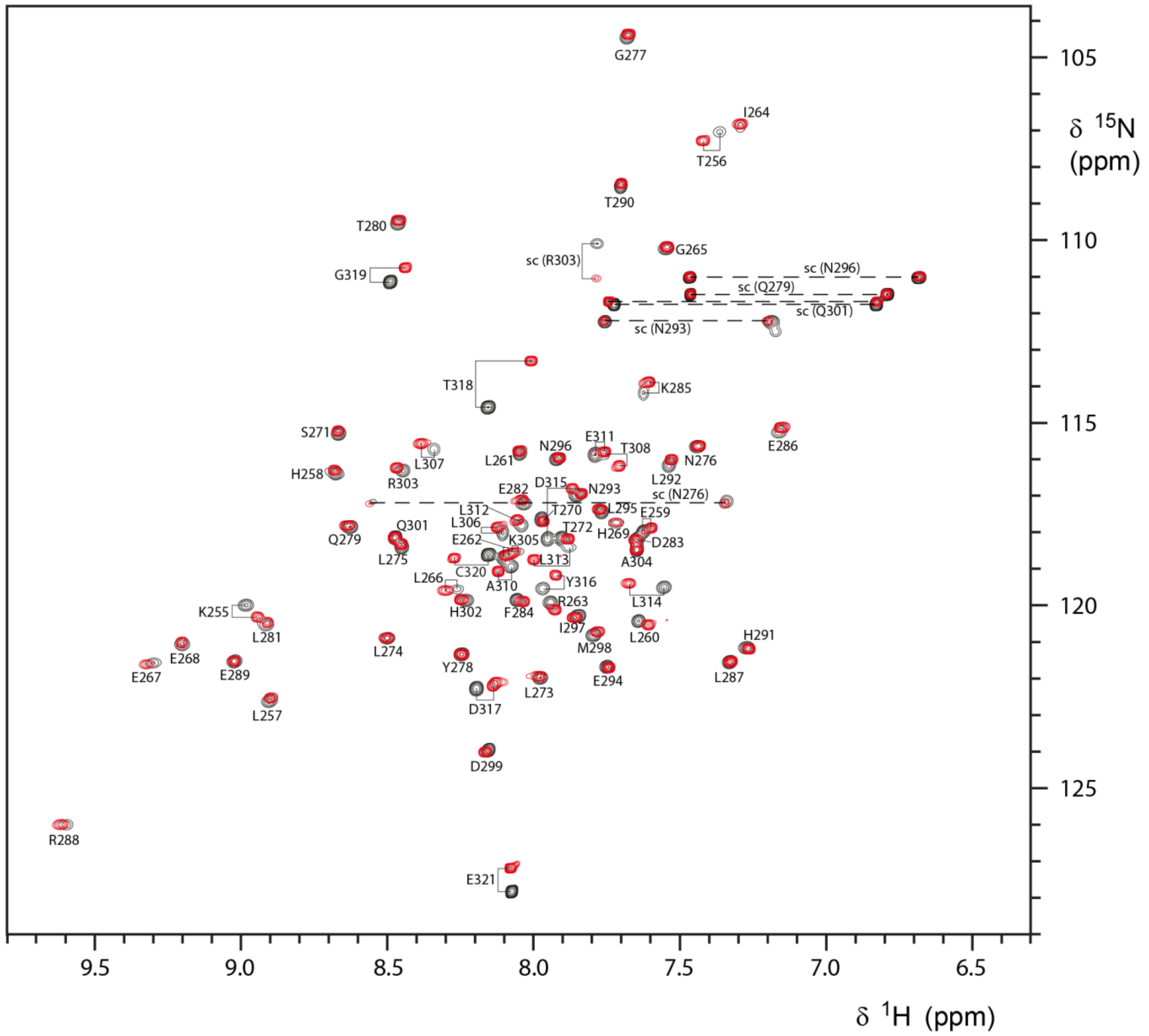

Supplementary Figure S6. Overlay of 2D ^{1}H-^{15}N HSQC spectra of SAM$_C$ under reducing and oxidizing conditions. 2D ^{1}H-^{15}N HSQC spectra of the disulphide bond-stabilized SAM$_C$ homodimer (red, oxidizing conditions) and unbridged SAM$_C$ (black, reducing conditions) have been recorded with 1.4 mM SAM$_C$ in 50 mM phosphate buffer, 20 mM NaCl, 0.2 mM EDTA (pH 6.4) at 35 °C. Assignment information is provided. Some resonances of the unbridged homodimer are broadened (T256, I264, L266, K285, R288, K305, L307, T308, A309, L313) because of chemical exchange (i.e., monomer-dimer equilibrium). Most of the backbone (H^N, N) correlations have minor differences in chemical shift between the two spectra. The largest chemical shift differences are observed for residues close to the oxidation site at C320 (T318, G319, C320, E321), to the C-terminus of helix α5 (L313, L314, D315) or to the N-terminus of SAM$_C$ (K255, T256). The apparent ^{15}N chemical shift difference of the aliased side chain (sc) signal of R303 is because different spectral widths were used in the two HSQC spectra.

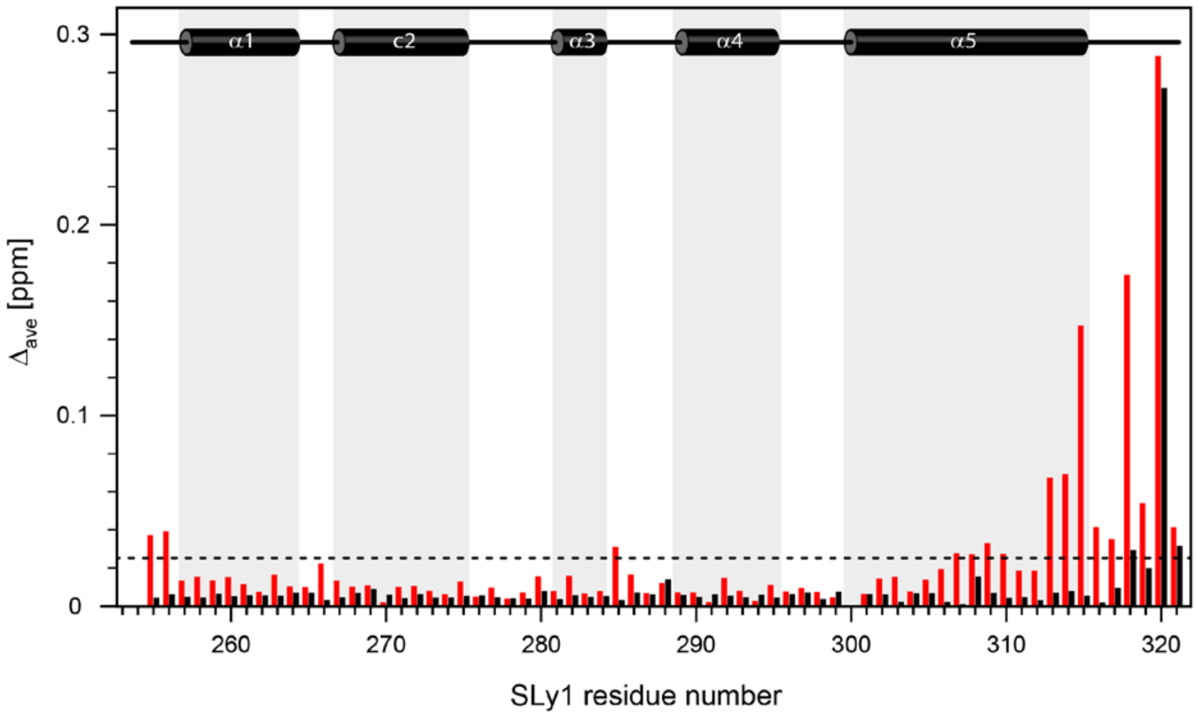

Supplementary Figure S7. Weighted average ^{1}H and ^{15}N backbone chemical shift changes of SLy1 SAM because of the S320C exchange and disulphide bond formation. Black bars represent the weighted average chemical shift differences (Δ_{ave}) in the 2D ^{1}H-^{15}N HSQC spectra of SAM_{wt} and SAM_{C} under reducing conditions. The largest shift is observed for the S320C exchange, with small shifts for sequence-neighbouring residues. Red bars show Δ_{ave} values between SAM_{wt} and SAM_{C} under oxidizing conditions, which cause SAM homodimer stabilization by intermonomer disulphide bond formation for SAM_{C}. The large Δ_{ave} for residue 320 is because of the S320C exchange. In addition, there are a few larger than average changes in the C-terminal region close to the mutation site, and both in the C-terminal part of helix α5 (residues 313–315) and at the N-terminus. The mean shift difference Δ_{ave} for each backbone (H^{N}, N) correlation between two 2D ^{1}H-^{15}N HSQC spectra was calculated according to $\Delta_{ave} = 0.5[(\Delta_{H})^{2} + (\Delta_{N}/5)^{2}]^{1/2}$ (Grzesiek *et al.* (1996) *Nat. Struct. Biol.* **3**, 340–345). The dotted line represents the average of all observed Δ_{ave} values for the oxidized state.

Supplementary Table S8. Structural statistics for the SLy1 SAM_C NMR structure ensemble.

**Completeness of resonance assignment**	
Protein, backbone[a] (337/346)	97.4%
Protein, all atoms[b] (788/827)	97.0%
**Experimental structure restraints**	
NOE distance restraints	
Unambiguously assigned	2,466
Intraresidue ($i = j$)	694
Interresidue (Intramonomer)	
Sequential ($\|i - j\| = 1$)	493
Medium Range ($1 < \|i - j\| < 5$)	637
Long Range ($\|i - j\| \geq 5$)	406
Intermonomer	236
Ambiguously assigned	1,593
H-bonds	16
Dihedral angles Chi1	24
Dihedral angles from TALOS+ (phi/psi)	58/58
**Evaluation of the 15 final low energy structures**	
Systematic NOE violations of more than 0.1 Å	0
Coordinate precision (Å)[c]	
Backbone heavy atoms	0.31 ± 0.08
All heavy atoms	0.57 ± 0.06
RPF score[d]	
DP-score	0.872
Recall	0.936
Precission	0.946
Ramachandran statistics (%)[e]	
Residues in most favoured regions	85.5 ± 1.4
Residues in additionally allowed regions	13 ± 1.3
Residues in generously allowed regions	0.8 ± 0.8
Residues in disallowed regions[f]	0.8 ± 0.8

[a] Obtained from the $^{1}H^{N}$, $^{13}C^{\alpha}$, ^{15}N, $^{13}C'$ and $^{1}H^{\alpha}$ resonances.
[b] Routinely assigned ^{1}H, ^{15}N and ^{13}C resonances are taken into account, excluding the N-terminal and Lys amino groups, guanidino groups of Arg, side chain hydroxyl protons of Ser, Thr, Tyr, thiol protons of Cys, carboxyl resonances of Asp and Glu, and the quarternary aromatic carbons. ^{1}H belonging to the same methyl group and Phe, Tyr $^{1}H^{\delta}$, $^{1}H^{\varepsilon}$ are counted as one signal.
[c] Root mean square deviation of all the structures from the average structure coordinates.
[d] Calculated RPF score according to Huang *et al.* (2012) *Nucleic Acids Res*. **40**, W542-W546.
[e] Calculated by the program PROCHECK-NMR (Laskowski *et al.* (1996) *J. Biomol. NMR* **8**, 477–486).
[f] Residues in the disallowed region are outside the structured core domain of SAM (L257–D315).

Supplementary Table S9. Hydrogen bonds and salt bridges across the dimer interface of the NMR structure of SAM_C and the X-ray structure of SAM_{wt}.

Monomer A			Monomer A'			Distance (Å)[a]	
SSE	Residue	Atom	Atom	Residue	SSE	NMR	X-ray
N-term	P254	[O]	[O$^{\eta}$]	Y316	*C-term*'	2.97	3.28
N-term	K255	[N$^{\zeta}$]	[OXT]	E321	*C-term*'	2.70	–
α1	R262	[N$^{\varepsilon}$]	[O$^{\delta1}$]	D315	α5'	2.62	3.54
α1	R262	[N$^{\varepsilon}$]	[O$^{\delta2}$]	D315	α5'	–	2.72
α1	R262	[N$^{\eta2}$]	[O$^{\delta1}$]	D315	α5'	–	3.46
α1	R262	[N$^{\eta1}$]	[O$^{\varepsilon1}$]	E311	α5'	2.99	–
α1	R262	[N$^{\eta1}$]	[O$^{\varepsilon2}$]	E311	α5'	3.44	–
α1	R262	[N$^{\eta2}$]	[O]	E311	α5'	–	3.55
α5	P300	[O]	[N$^{\varepsilon2}$]	Q301	α5'	2.91	2.91
α5	Q301	[N$^{\varepsilon2}$]	[O]	P300	α5'	2.91	2.91
α5	E311	[O]	[N$^{\eta2}$]	R262	α5'	–	3.55
α5	E311	[O$^{\varepsilon1}$]	[N$^{\eta1}$]	R262	α5'	2.99	–
α5	E311	[O$^{\varepsilon1}$]	[N$^{\eta1}$]	R262	α5'	3.44	–
α5	D315	[O$^{\delta1}$]	[N$^{\varepsilon}$]	R262	α1'	2.62	3.54
α5	D315	[O$^{\delta1}$]	[N$^{\eta2}$]	R262	α1'	–	3.46
α5	D315	[O$^{\delta2}$]	[N$^{\varepsilon}$]	R262	α1'	–	2.72
C-term	E321	[OXT]	[N$^{\zeta}$]	K255	*N-term*'	2.70	–
C-term	Y316	[O$^{\eta}$]	[O]	P254	*N-term*'	2.97	3.28

[a]Distances larger than the cutoff value of 3.6 Å are indicated by a hyphen.

Supplementary Table S10. X-ray data collection and refinement statistics for SLy1 SAM_{wt}.

**Data acquisition**	
Beamline/ Detector	ID30A/Eiger X 4M
Wavelength λ (Å)	0.9677
Resolution range (Å)	47.34–2.05 (2.11–2.05)
Space group	$P\ 4_1\ 2_1\ 2$
Unit cell a, b, c (Å)<br>α, β, γ (°)	44.09 44.09 94.67<br>90 90 90
Total reflections	80,277 (6,533)
Unique reflections	6,358 (481)
Multiplicity	12.6 (13.6)
Completeness (%)	99.9 (100.00)
Mean I/δ(I)	13.9 (2.1)
Wilson B-factor ($Å^2$)	55.8
R-merge	0.092 (1.281)
R-meas	0.096 (1.330)
R-pim	0.027 (0.355)
CC (1/2)	0.999 (0.381)
**Refinement**	
Resolution range in refinement (Å)	39.97–2.05 (2.124–2.05)
Reflections used in refinement	6,318 (604)
Reflections used for R-free	319 (29)
R-work	0.2183 (0.3766)
R-free	0.2421 (0.3764)
Number of non-hydrogen atoms	547
macromolecules	537
solvent	10
Protein residues	65
RMS(bonds)	0.008
RMS(angles)	1.22
Ramachandran favored (%)	96.83
Ramachandran allowed (%)	3.17
Ramachandran outliers (%)	0.00
Rotamer outliers (%)	1.67
Clashscore	7.63
Average B-factor $Å^2$	65.05
macromolecules	65.14
solvent	60.30
Number of TLS groups	4

Supplementary Table S11. Equilibrium dissociation constants (*K_d*) of SAM domain homodimers.

SAM variant	K_d of dimerization [µM]	reference
EphA4 SAM	500–5,000	Stapleton *et al.* (1999) *Nat. Struct. Biol.* **6**, 44–49.
Ste11 SAM	~500	Grimshaw *et al.* (2004) *J. Biol. Chem.* **279**, 2192–2201.
SLy1 SAM_{wt}	117 (33, 423)	this manuscript, AUC data
SLy1 SAM_{wt}	153 ± 25	this manuscript, MST data
SLy1 SAM_{lg}	2.2 (1.8, 2.6)	this manuscript, AUC data
SLy1 SAM_{lg}	5.4 ± 1.4	this manuscript, MST data
